# Room-Temperature Response Performance of Coupled Doped-Well Quantum Cascade Detectors with Array Structure

**DOI:** 10.3390/nano13010110

**Published:** 2022-12-26

**Authors:** Jie Chen, Fengwei Chen, Xuemin Wang, Yunhao Zhao, Yuyang Wu, Qingchen Cao, Tao Jiang, Keyu Li, Yang Li, Jincang Zhang, Weidong Wu, Renchao Che

**Affiliations:** 1 Laboratory of Advanced Materials, Shanghai Key Lab of Molecular Catalysis and Innovative Materials, Academy for Engineering & Technology, Fudan University, Shanghai 200438, China; 2 Laser Fusion Research Center, China Academy of Engineering Physics, Mianyang 621900, China; 3 Zhejiang Laboratory, Hangzhou 311100, China

**Keywords:** QCD, responsivity, energy level interactions, electron concentration, doping

## Abstract

Energy level interaction and electron concentration are crucial aspects that affect the response performance of quantum cascade detectors (QCDs). In this work, two different-structured array QCDs are prepared, and the detectivity reaches 10^9^ cm·Hz^1/2^/W at room temperature. The overlap integral (OI) and oscillator strength (OS) between different energy levels under a series of applied biases are fitted and reveal the influence of energy level interaction on the response performance. The redistribution of electrons in the cascade structure at room temperatures is established. The coupled doped-well structure shows a higher electron concentration at room temperature, which represents a high absorption efficiency in the active region. Even better responsivity and detectivity are exhibited in the coupled doped-well QCD. These results offer a novel strategy to understand the mechanisms that affect response performance and expand the application range of QCDs for long-wave infrared (LWIR) detection.

## 1. Introduction

Compared with multiquantum-well infrared photodetectors (QWIPs), quantum cascade detectors (QCDs) are characterized by the insertion of asymmetric, multilevel, undoped layers as transport regions (TRs) between doped layers [1,2,3,4]. This special structure allows photoelectrons to migrate from one period to the next by longitudinal optical (LO) phonon-assisted tunneling instead of an applied bias to bend the barrier-well structure [5,6,7,8,9]. As an ideal photovoltaic detector without dark current, QCDs have attracted widespread attention over the last decade [10,11]. Owing to the mature preparation and stable Si+ doping technology, AlGaAs/GaAs has become the most prevalent material in QCDs [12,13,14]. The absorption wavelength and global transition rate of photoelectrons can be adjusted by controlling the thickness of the doped layers and the Al concentration, which has been proven theoretically and experimentally [15,16]. The patch-antenna structure and quantum dot QCDs based on AlGaAs/GaAs have been recently fabricated [17,18,19,20]. However, exploring improvements to the QCD detection performance has not been sufficiently investigated, and improving absorption and extraction efficiency is still an urgent requirement.

The electron migration behaviors of complex multiquantum wells in QCDs have been recently described theoretically [16,21,22]. These studies show that the energy level interaction and electron concentration play a key role in the absorption and extraction efficiency, which are closely related to the QCD response performance. For example, as the electron concentration triples, the responsivity of the QCD improves from 2.5 to 10.7 mA/W, and the detectivity increases by an order of magnitude at low temperatures [23]. Nevertheless, when the doping concentration *n_s_* reaches a critical value, the quantum efficiency saturates. Then, the detectivity of the QCD will decrease with further increased doping [24]. Increasing the thickness of the doped layer improves the carrier concentration, which serves as a simple and efficient way to enhance the quantum efficiency [25,26,27]. Recently, a coupled doped-well structure was proposed and fabricated where the active region (AR) consisted of two doped layers with the same intrinsic parameters [15,28]. The responsivity of the device reached 60 mA/W at 50 K under 0 V and rose to 100 mA/W under −2 V [28]. On the other hand, energy level splitting gives the QCD device broadband absorption. However, the effect of the energy level interaction and electron concentration on the response performance of the detector has not been fully studied experimentally. In addition, the array of coupled doped-well QCDs has not been reported so far.

In this work, two different-structured array QCDs are prepared by standard UV photolithography and the wet-etching technique. These QCD devices show a peak responsivity at 15.4 μm and exhibit a detectivity of 10^9^ cm·Hz^1/2^/W at room temperature. Compared to traditional QCD, the coupled doped-well structure exhibits a higher responsivity and detectivity. Energy level interaction is simulated to reveal the effect of applied bias on the detection property of QCD devices. In addition, the electron distribution characteristic is discussed qualitatively. By combining the results of the experiment and simulation, the coupled doped-well QCD can serve as a powerful candidate structure to expand the application of QCD devices.

## 2. Experimental Details

Two Al_0.28_Ga_0.72_As/GaAs QCD structures (single well and coupled doped well) are grown on GaAs substrate via solid-source molecular beam epitaxy (MBE) at 605 °C. Both GaAs and AlGaAs have growth rates of 1 μm/h. To ensure the planer and vertical uniformity of the single layer thickness in the active region, the substrate rotation speed is set to ~30 rpm considering the angle of beam flux and the substrate surface. The growth sequence comprised a 400 nm thick GaAs buffer layer doped to 2.5 × 10^18^ cm^−3^, followed by a core region with 30 periods of asymmetric well–barrier multilayers, and finally a 200 nm thick GaAs contact layer doped to 2.5 × 10^18^ cm^−3^. The core regions in Angstroms for one period are 58/**99**/60/33/41/47.5/50/50 (single-well QCD (S-QCD)) and 58/**99**/33/**99**/60/33/41/47.5/50/50 (coupled doped-well QCD (C-QCD)), respectively. The underlined thickness values correspond to Al_0.28_Ga_0.72_As barriers, while the bold values indicate the uniformly doped layer (n-type, 2 × 10^17^ cm^−3^).

**Figure 1:**
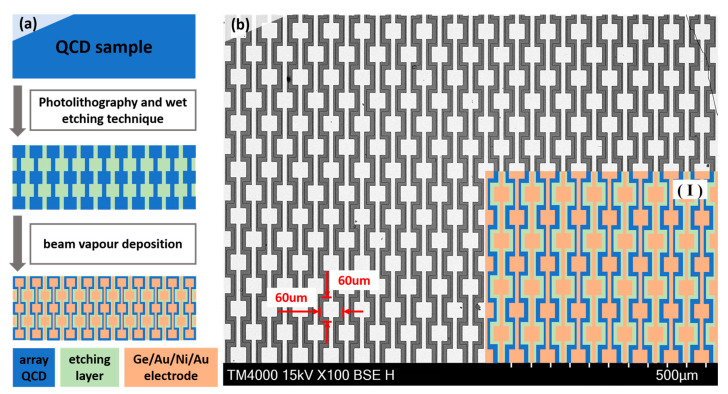
Array QCD structure. (**a**) The preparation steps of the array QCD. (**b**) The SEM of array QCD. The area of each unit is 60 × 60 μm, as marked by the red ruler. Inset (Ⅰ) is the corresponding structure diagram of the array unit.

To optimize the light incident mode and increase the absorption of the structure compared to the facet, an array structure was fabricated by standard UV photolithography and the wet-etching technique, as shown in Figure 1a,b. The whole structure consists of 30 × 56 array units, and the area of each unit is 60 × 60 μm. Then, the top and bottom electrodes, as a photoelectron transmitter, were deposited by the electron beam evaporation of the Ge/Au/Ni/Au electrode. Every array unit was electrically connected by a 10 μm wide electrode to ensure the collection of photoelectrons. Thermal annealing (390 °C for 30 s) was adopted under a nitrogen atmosphere to provide good ohmic contact.

The electron wave function in the cascade structure (including energy levels interaction and carrier redistribution) is obtained by solving the Schrodinger–Poisson equation with an effective 2-band Kane model [29]. The structural parameters of the simulation are described above, and the temperature is set to 300 K. Electrons follow the Fermi–Dirac distribution. Finally, 3 periods of the cascade structure were calculated, but only the central 1 period is depicted.

## 3. Results and Discussion

### 3.1. Normal Incident Irradiation

**Figure 2:**
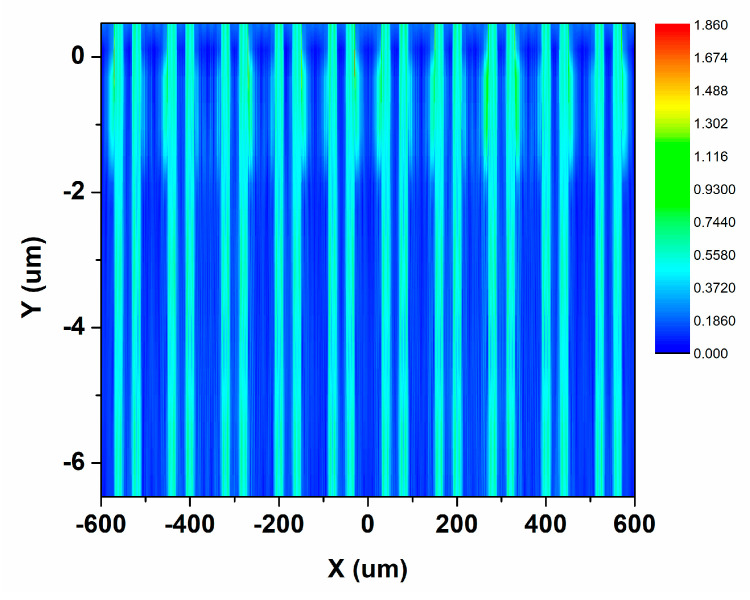
Cross-sectional distribution of the light field in the QCD device. The Y-axis coordinate represents the thickness of the device and the substrate, where zero is the surface of the QCD devices.

Due to the array structure and electrode, two absorption ways are mainly considered in this work. One is the random scattering through the edge of the metal, and the other one is through the surface plasma resonance of periodic metal structures. The latter primarily uses the interaction between incident photons and electrons at the metal interface to couple light into the QCD. Figure 2 shows the cross-sectional distribution of the light field calculated by FDTD in the QCD device. The cascade structure thickness of the S-QCD and the C-QCD is 1.32 μm and 1.71 μm, respectively, corresponding to the regions in the simulation result (Y-axis: −2~0 μm). The result indicates that the normal incident irradiation is coupled into the cascade structure.

### 3.2. Microstructure and Band Structure

**Figure 3:**
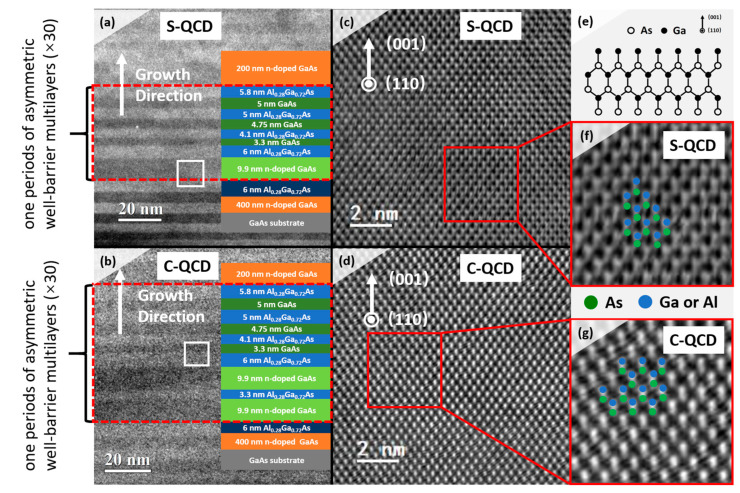
Microstructure and atomic arrangement. Low-magnification TEM image of (**a**) S-QCD and (**b**) C-QCD. The dashed red frame represents one period of the asymmetric well–barrier multilayers. The white arrows denote the growth direction, while the white frame shows the corresponding regions of (**c**,**d**), which correspond to samples S-QCD and C-QCD, respectively. Cross-sectional HRTEM image of (**c**) S-QCD and (d) C-QCD. (**e**) Atomic arrangement diagram of the GaAs sphalerite structure. Cation–anion pairs of AlGaAs in (**f**) S-QCD and (**g**) C-QCD.

The cascade structures were observed using transmission electron microscopy (TEM) and illustrated by the color diagram, as shown in Figure 3a,b. The contrast intensity between the GaAs and AlGaAs layer indicates the alternating growth of the well and barrier in low magnifications. To obtain precise constructions, the white frame regions in Figure 3a,b are confirmed by cross-sectional high-resolution transmission electron microscopy (HRTEM) in Figure 3c,d, respectively. Although the region includes well and barrier layers, there is no lattice mismatch observed at the interface between the well–barrier layer due to the nearly uniform lattice constants (5.653 Å for GaAs and 5.658 Å for Al_0.28_Ga_0.72_As) [30]. Meanwhile, the HRTEM shows an excellent growth quality without distinct defects, such as dislocations and distortions. The cation–anion pairs of Ga+ (Al+) and As- are marked in Figure 3f,g based on the atomic arrangement diagram of the GaAs sphalerite structure [31,32,33].

**Figure 4:**
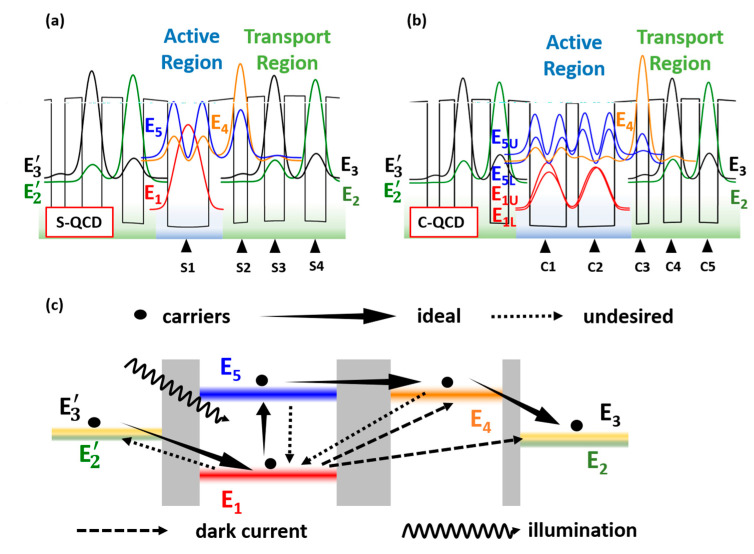
Band structures and schematic diagram of the QCD. Conduction band structures and wave functions of samples (**a**) S-QCD and (**b**) C-QCD. The colored background represents the active region (blue) and transport region (green). (**c**) Schematic diagram and migration of electrons in the cascade structure.

Figure 4a,b shows the sub-band structures and electron wave function of each quantum cascade structure. In the bound-to-bound structure, E_1_ and E_5_ serve as the initial and final states of the electron absorption photon transitions, respectively, while the single electron state of E_4_, serves as an extractor, it has a strong overlap integral with E_5_ [15]. The E_3_ and E_2_ function as transfer stations and provide migration paths for electrons with LO-phonon scattering [34]. The inserted doped layer in the C-QCD complicates the sub-band energy level in the AR. As the two doped wells have the same intrinsic characteristics, the energy levels at the same state will split [35]. Consequently, the excited state E_5_ (ground state of E_1_) will split into upper excited level E_5U_ and lower excited level E_5L_ (upper ground level E_1U_ and lower ground level E_1L_), as shown in Figure 4b. Figure 4c shows the schematic structure and migration path of the QCD. At thermal equilibrium, carriers mostly populate at the ground state E_1_ [36,37]. Under illumination, electrons will transition to the excited state E_5_ due to the high oscillator strength between E_1_ and E_5_ [38,39,40]. A few electrons will diagonally migrate to other energy levels under thermodynamic equilibrium [15]. However, the electron transition rate is lower than vertical migration by several orders of magnitude [16,21]. The extractor level E_4_ should have a strong OI to the excited energy level E_5_ to achieve a high extraction efficiency based on resonant tunneling [41,42]. A slight deviation in the OI between E_5_ and E_4_ can be compensated by the applied bias. The energy separations in the two quantum cascade structures are listed in Table 1.

### 3.3. Response Performance and Detectivity

**Figure 5:**
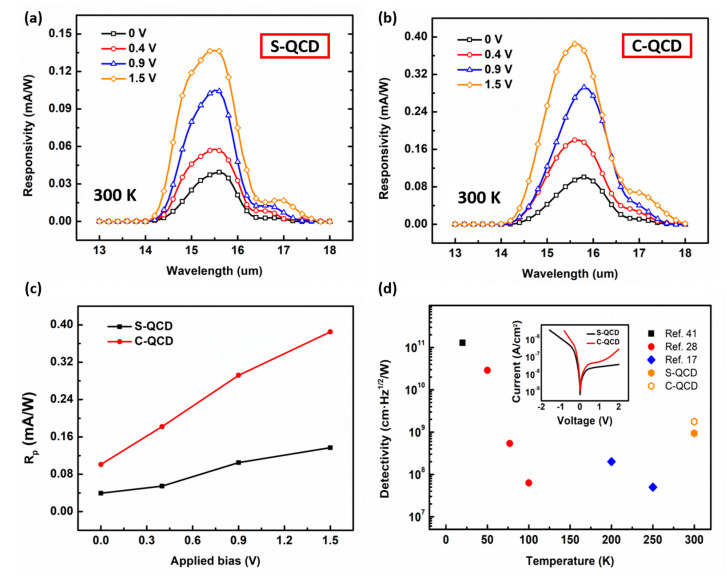
Response performance and detectivity. The responsivity of (**a**) S-QCD and (**b**) C-QCD under different applied biases. (**c**) Peak responsivity (*Rp*) of the two QCDs as a function of the applied bias at 300 K. (**d**) Detectivity D* of the two QCD devices at 300 K. Inset: current density as a function of the bias under the illumination by a blackbody at 300 K.

The responsivity of the two QCDs with normal incident irradiation under different biases was tested, as shown in Figure 5a,b. The absorption peak of the S-QCD is located at 15.4 μm, which agrees with the original design. In contrast, the absorption peak of the C-QCD is located at 15.8 μm with a wider response spectrum. This is due to the coupling between energy levels which results in the splitting of the ground state and excited state levels. The peak responsivity *(Rp)* values of the two QCD devices with different applied biases are demonstrated in Figure 5c. The *Rp* rises with the applied bias increasing, which is in agreement with that reported previously [17,28]. The upward trend of the C-QCD is significantly higher than that of the S-QCD. This can be attributed to the introduction of the additional doped layer that optimizes the energy level alignment and increases the electron concentration in the active region, thereby improving the responsivity of the device.

To further compare the response performance of the two structures, the detectivity D* of the two QCDs is calculated as follows:

(1) $$D^{*}=R\left(\lambda \right)\sqrt{\frac{R_{0}A}{4k_{B}T}}$$

where *R(λ)* is the responsivity, and *k_B_* and *T* are the Boltzmann constant and temperature, respectively. The inset of Figure 5d exhibits the current densities versus bias under illumination by a blackbody at 300 K. The resistance *R*_0_, as a key parameter that governs the Johnson noise of the device, has been calculated by deriving from the slope of the V-I curves. Hence, the D^*^ of the two QCD devices is obtained under room temperature and zero bias, as shown in Figure 5d. The detectivity of the S-QCD and the C-QCD is 9.39 × 10^8^ cm·Hz^1/2^/W and 1.76 × 10^9^ cm·Hz^1/2^/W at 0 V and 300 K, respectively. Figure 5d also shows the detectivity for other temperature points in reference. Generally, the detectivity decreases with increasing temperature [24]. However, the QCD in this work still maintains a high detectivity at room temperature and the detectivity of the C-QCD is higher two times than that of the S-QCD.

### 3.4. Energy Level Interaction

**Figure 6:**
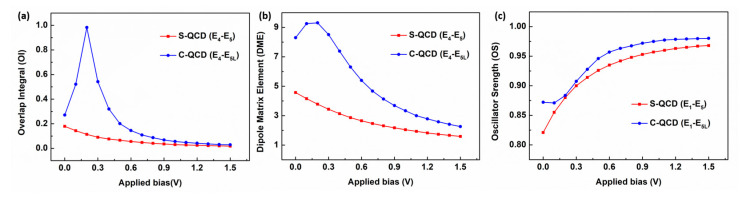
Correlations between different energy levels. (**a**) OI and (**b**) DME over the applied bias between the excited state (E_5_) and extractor energy level (E_4_). (**c**) Oscillator strength between the ground state (E_1_) and excited state (E_5_).

Several experimental results have shown that QCDs can exhibit much higher response performance under small biases [17,28]. This is ascribed to the detector performance of the QCD being closely related to the absorption and extraction efficiency of photoelectrons. While the applied bias plays an important role in the migration of photoelectrons between different energy levels [43]. Therefore, a series of dipole matrix elements (DMEs), OIs, and OSs under different applied biases are simulated to qualitatively reflect this rule, as shown in Figure 6.

The extraction efficiency relies primarily on the correlations between the excited level and the extractor level. Therefore, the OI and DME between E_5_ and E_4_ are considered in the S-QCD. In the C-QCD, there is a stronger coupling between the lower excited level and extractor level (Figure 4b), which is also the main migration path for photoelectrons, especially under applied bias. So, only the OI and DME between E_5L_ and E_4_ are considered in the C-QCD. With the applied bias increasing and changing the initial optimization energy level arrangement, the DME and OI of the S-QCD decrease, as shown in Figure 6a,b. Compared with S-QCD, the DME and OI of the C-QCD first increase and then decrease. This can be ascribed to the characteristics of the coupled doped well, which cause the excited state E_5_ to split to the upper excited level E_5U_ and lower excited level E_5L_. At zero bias, the structural design positions the extractor energy level E_4_ just between E_5U_ and E_5L_ (Figure 4b). With the applied bias increasing, the lower excited level and extractor level overlap and then separate. Consequently, the OI and DME of the C-QCD exhibit a process of rising and then falling, meanwhile showing a stronger extraction efficiency compared to the S-QCD. In addition to affecting the arrangement between adjacent energy levels, the applied bias also changes the oscillator strength, which is closely related to the photoelectric conversion efficiency. Therefore, the oscillator strengths between the ground state (E_1_) and excited state (E_5_) in the AR are calculated. Since the electrons on both the upper and lower ground levels transition to E_5L_ in the C-QCD, E_1_ includes E_1L_ and E_1U_. With the applied bias increasing, the OS in both the S-QCD and the C-QCD increases and then reaches saturation, while a higher value is shown in the latter. Consequently, the applied bias is beneficial to improve the response performance, and the extraction efficiency of photoelectrons can be optimized by using the coupled doped-well structure.

### 3.5. Redistribution of Electrons

In addition to the applied bias, the detection efficiency is also affected by electron concentration. Generally, the doping carriers are ideally distributed in the active region, however, the thermal diffusion will cause the actual distribution of carriers to deviate from the ideal state and redistribute. Therefore, the electron density distribution in real space in the two QCDs at room temperatures has been simulated.

The electron density at room temperature in the different wells is listed in Table 2. The position of each quantum well corresponds one-to-one to the quantum wells in Figure 4a,b. Ideally, electrons are mainly distributed in the doped well (~2 × 10^11^ cm^−2^ in S1, C1, and C2). However, at room temperature, the concentration of carriers in the doped well will decrease and diffuse into the adjacent well in the transport region. This is attributed to the thermal effects, which lead to the mutual diffusion of electrons [44]. Note that, owing to the two doping layers, the carrier concentration in the active region of the C-QCD is higher than that in the S-QCD at room temperature. Since the absorption efficiency is proportional to the carrier concentration, there is a higher response performance in the C-QCD.

## 4. Conclusions

In this work, two different array quantum cascade detectors are fabricated. The array QCDs exhibit a higher detectivity of 10^9^ cm·Hz^1/2^/W at room temperature; meanwhile, the coupled doped-well QCD shows better responsivity and detectivity. Moreover, the interactions between different energy levels in the cascade structures are considered. The results demonstrated the energy level interaction can be optimized by the applied bias, which reveals the essence of the higher absorption and extraction efficiency in the coupled doped-well QCD. In addition, the electrons are redistributed due to the thermal effect, and the coupled doped-well QCD shows a higher electron concentration at room temperature. Introducing an extra doped layer can compensate for the carrier concentration decreasing in the active region caused by thermal diffusion. These results indicate that the coupled doped well can serve as a powerful candidate structure for improving response performances of QCD devices, and can also be applied to focal-plane array QCDs.

## Figures and Tables

**Table 1:** Energy separations in the two QCD devices.

**S-QCD**	**E_1_–E_5_**		**E_4_–E_5_**		**E_2_–E_4_**		**E_2_–E_4_**	
	81 meV		6 meV		29 meV		36 meV	
**C-QCD**	**E_1L_–E_5L_**	**E_1L_–E_5U_**	**E_1U_–E_5L_**	**E_1U_–E_5U_**	**E_4_–E_5L_**	**E_4_–E_5U_**	**E_3_–E_4_**	**E_2_–E_4_**
	78 meV	89 meV	75 meV	86 meV	3 meV	8 meV	30 meV	36 meV

**Table 2:** Electron density in each well of two QCDs.

**Sample**	**Single-Well QCD**			
Well	S1	S2	S3	S4
Electron density (cm^−2^)	1.45 × 10^11^	0.08 × 10^11^	0.2 × 10^11^	0.23 × 10^11^
**Sample**	**Coupled Doped-Well QCD**			
Well	C1	C2 C3	C4	C5
Electron density (cm^−2^)	1.68 × 10^11^	1.7 × 10^11^ 0.09 × 10^11^	0.24 × 10^11^	0.29 × 10^11^

## Data Availability

Not applicable.
